# Molecular and immune-mediated resistance to antibody-drug conjugates in breast cancer

**DOI:** 10.3389/fimmu.2026.1927515

**Published:** 2026-10-09

**Authors:** Alvise Mattana, Chiara Veronese, Julia Penitenti, Elettra Unti, Elena Varotti, Andrea Vingiani, Daniele Lorenzini, Roberto Boffi, Agnese Graziosi, Romano Danesi, Giancarlo Pruneri

**Affiliations:** 1 Department of Advanced Diagnostics, Fondazione IRCCS Istituto Nazionale dei Tumori, Milan, Italy; 2 Department of Experimental and Clinical Biomedical Sciences, University of Florence, Florence, Italy; 3 Pathology Unit, ARNAS Civico di Cristina Benfratelli Hospital, Palermo, Italy; 4 Pathology Unit, ASST Cremona, Cremona, Italy; 5 Oncology and Hematology-Oncology Department, University of Milan, Milan, Italy

**Keywords:** antibody-drug conjugates (ADC), biological mechanism, breast cancer, immunological mechanism, resistance

## Abstract

Antibody-drug conjugates (ADCs) have progressively advanced the conceptual framework of targeted oncology, evolving from a way to deliver chemotherapy selectively to tumor cells into drugs that also interact with the immune system to support their action. In breast cancer, the approval of several novel ADCs has transformed the therapeutic landscape across HER2-positive, HER2-low and triple-negative disease. Despite these advances, primary and acquired resistance remain the dominant clinical challenge. This narrative review classifies these resistance mechanisms, examining their interactions and therapeutic implications. We organize resistance along two highly interconnected domains. A molecular domain encompasses target antigen alterations, defective endolysosomal trafficking, payload efflux, and altered apoptotic thresholds. An immunological domain includes impaired Fc-mediated functions, escape from immunogenic cell death, and microenvironment-mediated immunosuppression. These domains frequently converge each other to drive clinical failure. Importantly, immune-mediated effects depend heavily on specific ADC constructs, and their clinical impact requires further validation. While routine assays currently assess baseline candidate markers, advancing rational therapeutic sequencing will require validated biomarkers capable of capturing dynamic processes, such as receptor trafficking or functional immune evasion.

## 1 Introduction

“We have to learn how to aim chemically”. With this statement, Paul Ehrlich, Nobel Prize in Physiology or Medicine in 1908, revolutionized therapeutics through his “magic bullet” theory, which culminated in the development of Salvarsan, an early targeted antimicrobial chemotherapeutic agent against syphilis (1).

Although Ehrlich originally established the concept of chemotherapy in an infectious disease context, the paradigm eventually expanded into oncology with the advent of cytotoxic cancer therapy. This new era operated on the principle that rapidly dividing tumor cells are highly vulnerable to agents that interfere with DNA replication, nucleotide metabolism, or microtubule dynamics. Early milestones included the use of nitrogen mustards for lymphoma in the 1940s and the subsequent development of antimetabolites, highlighted by Sidney Farber’s use of the folic acid antagonist aminopterin to achieve remission in childhood leukemia (2). While these conventional therapies established the foundation of medical oncology, their indiscriminate cytotoxicity and the inevitable emergence of drug-resistant tumors underscored the critical need for more targeted approaches, ultimately paving the way for the development of antibody-drug conjugates (ADCs) (3, 4).

Despite the development of all these pharmacological strategies, which are still widely used in daily clinical practice, have important limitations: they are not sufficiently selective and affect healthy tissues, potentially causing serious adverse events. For this reason, a new generation of cancer treatments was emerged. Indeed, while not exhaustive, three different approaches particulary relevant to ADC development were developed: small molecule inhibitors, monoclonal antibodies and immunotoxins (5, 6).

Small molecule inhibitors target intracellular kinases involved in tumor growth and survival. The approval of imatinib in 2001, the first kinase inhibitor in oncology, marked the beginning of the precision oncology era (5). In contrast, monoclonal antibodies (mAbs) recognize tumor-associated antigens at the cell surface and engage anti-tumor activity primarily through receptor-pathway inhibition and Fc-mediated immune effector functions, rather than through direct cytotoxicity (7, 8). Finally, immunotoxins were designed to deliver cytotoxic proteins selectively to malignant cells by chemically linking whole bacterial or plant toxins to antibodies, or through genetically engineered recombinant constructs fusing tumor-selective ligands to modified toxins (9–11).

Antibody-drug conjugates (ADCs) emerged as the natural evolution of this design rationale, combining the selectivity of monoclonal antibodies in recognizing tumor antigens with the cytotoxic potency of conventional chemotherapy (12). ADCs also act as immunomodulatory agents, integrating direct cytotoxicity with immunogenic cell death, Fc-mediated effector functions, and tumor microenvironment remodeling.

Breast cancer represents the paradigmatic setting for ADCs developed in solid cancer. Since the FDA approved trastuzumab emtansine (T-DM1) in 2013 (13),more ADCs have entered routine clinical use, yet the “*Ehrlichian goal*” of selective chemical targeting has revealed itself to be more layered than originally envisioned: antibody selectivity now coexists with a complex immunological dimension, and resistance emerges from both fronts. This narrative review revisits ADC biology in breast cancer through this dual lens, integrating molecular and immunological axes of resistance.

## 2 ADC architecture

The three principal components of ADCs are the antibody, linker and payload.

### 2.1 Antibody component and Fc-mediated functions

The goal of monoclonal antibodies (mAbs) in ADCs is to serve dual functions: both as drivers that deliver cytotoxic agents into tumor cells and as immune effector platforms. Consequently, an ideal mAb must meet specific intrinsic requirements: high specificity and affinity for the molecular target, efficient cellular internalization, minimal expression on normal tissues, favorable pharmacokinetics with prolonged circulation half-life, and retention of Fc-mediated effector functions (14, 15). All currently approved breast cancer ADCs are based on humanized or fully human IgG1 mAbs. The IgG1 isotype is preferred over IgG2 and IgG4 because of its high affinity for the activating Fcγ receptors and its strong capacity to engage antibody-dependent cellular cytotoxicity (ADCC), antibody-dependent cellular phagocytosis (ADCP), and complement-dependent cytotoxicity (CDC), whereas IgG2 and IgG4 are poor inducers of the complement cascade and IgG4 additionally undergoes Fab-arm exchange that compromises effector function (16).

The conjugation of cytotoxic payloads does not substantially impair these Fc-mediated effector functions, which operate at the cell surface (17). The mechanistic details of these immunological functions are developed in section 3.1.1.

### 2.2 Linker technologies

The linker connects the antibody to the cytotoxic payload and is a critical determinant of ADC stability, drug release kinetics, and therapeutic efficacy. An ideal linker must remain stable in the systemic circulation to prevent premature release of the payload, which would otherwise cause off-target systemic toxicity and reduce the amount of drug effectively delivered to the tumor (18). The number of cytotoxic agents linked to each antibody influences the efficacy of the treatment. This ratio is defined as the Drug-to-Antibody Ratio (DAR). The favorable DAR range of 2 to 6 described by Sun et al. derives from specific analyses of antibody-maytansinoid conjugates and should not be presented as a universal ADC threshold, as DAR effects heavily depend on payload hydrophobicity, conjugation site, product heterogeneity, and linker design (19).

Finally, linkers are classified into two categories:

• Non-cleavable linkers (e.g., utilizing SMCC as the conjugation reagent to form the MCC linker moiety in the final trastuzumab-MCC-DM1 conjugate) release the cytotoxic agent only after complete lysosomal proteolysis of the antibody. This mechanism ensures high plasma stability and reduces off-target adverse events, while limiting the bystander effect, as the released catabolite is typically membrane-impermeant (13, 20). Indeed, T-DM1 employs the non-cleavable MCC linker, demonstrating excellent stability with a DAR of 3.5 (13).
• Cleavable linkers release the cytotoxic agent intracellularly through specific triggers. Protease-cleavable linkers, hydrolyzed by lysosomal cathepsins B and L, are exemplified by the GGFG tetrapeptide linker of trastuzumab deruxtecan (T-DXd), with a high DAR of approximately 8 (21). In contrast, Sacituzumab govitecan (SG), utilizes a hydrolyzable CL2A linker. This linker is not fully stable at physiologic pH; it spontaneously releases the SN-38 payload in serum with a half-life of approximately one day. Therefore, its drug release mechanism should not be considered confined exclusively to acidic lysosomal or tumor compartments, contributing uniquely to both its bystander effect and its systemic toxicity profile (22).

### 2.3 Cytotoxic payloads

Payloads are highly potent cytotoxic molecules that, once released intracellularly within endosomes or lysosomes, lead to cell death. Historically, these agents have primarily consisted of anti-tubulin agents (e.g., auristatins, maytansinoids) that inhibit mitotic spindle formation, or DNA-damaging agents. The payloads utilized in ADCs are significantly more potent than conventional chemotherapeutic drugs, exhibiting sub-nanomolar or even picomolar *in vitro* cytotoxicity, compared to the micromolar concentrations typical of standard agents (18).

A critical feature of modern payloads is the bystander effect. When a released payload is capable of diffusing out of the primary target cell into the interstitial space, it can be taken up by neighboring neoplastic cells, regardless of their antigen expression levels. While bystander killing may improve therapeutic activity in antigen-heterogeneous tumors (antigen-low or antigen-negative cells), it may also reduce selectivity and contribute to systemic toxicity. Furthermore, bystander activity depends on the chemical properties of the released species, membrane permeability, extracellular stability, local exposure, and tumor architecture; consequently, linker cleavability alone is insufficient to predict it (23).

Beyond traditional anti-mitotic and DNA-targeting agents, topoisomerase I inhibitors are now recognized as an established payload class in breast cancer. However, current research expanding into RNA polymerase inhibitors, immune-active payloads (immunomodulators), and degrader conjugates (PROTACs) remains experimental. Furthermore, the evolution of ADC design aims to overcome tumor resistance through dual-loading systems. For instance, recent developments include ADCs simultaneously conjugating a topoisomerase I inhibitor (exatecan) and an RNA polymerase II inhibitor (triptolide). While this ‘dual-killing’ strategy has demonstrated greater efficacy in patient-derived xenograft (PDX) models compared to monotherapies, this observation does not by itself demonstrate the prevention of acquired resistance (24).

## 3 ADC pharmacology

The pharmacology of ADCs reflects their hybrid nature, combining the behavior of a biologic antibody with that of a cytotoxic agent. This is expressed through their pharmacodynamics and pharmacokinetics, which must account for multiple circulating species, from the intact conjugate to the released free payload.

### 3.1 Pharmacodynamics

ADCs exert their therapeutic effect through two complementary modalities: direct cytotoxicity and immunological activation.

The direct cytotoxic action begins with the recognition and binding of the target antigen (e.g. HER2, TROP2) by the Fab region of the antibody. Following target engagement, the conjugate typically undergoes receptor-mediated endocytosis through the endolysosomal network. However, this canonical pathway is not uniform; internalization rates, receptor recycling kinetics, target shedding, endosomal sorting, and lysosomal delivery efficiency vary substantially across distinct ADC architectures and tumor types. Furthermore, while the classical route necessitates complete intracellular processing, alternative mechanisms (such as extracellular linker cleavage) can also operate to release the payload prematurely or in the tumor microenvironment, depending on the specific conjugate and its linker stability. Within the lysosomal compartment, the acidic environment and proteolytic enzymes activate the linker mechanism: in cleavable linkers, lysosomal proteases (cathepsins B and L) hydrolyze the peptide bond, releasing the free cytotoxic payload into the cytoplasm; in non-cleavable linkers, complete proteolytic degradation of the antibody backbone is required to liberate the active payload-amino acid catabolite. The released payload then engages its molecular target, microtubules in the case of maytansinoids (DM1), DNA topoisomerase I in the case of DXd and SN-38, ultimately triggering apoptotic cell death (23, 25).

Beyond this direct cytotoxicity, ADCs also act as immunomodulatory agents through three interconnected mechanisms (**Figure 1**): Fc-mediated effector functions, the induction of immunogenic cell death, and the remodeling of the tumor microenvironment.

**Figure 1:**
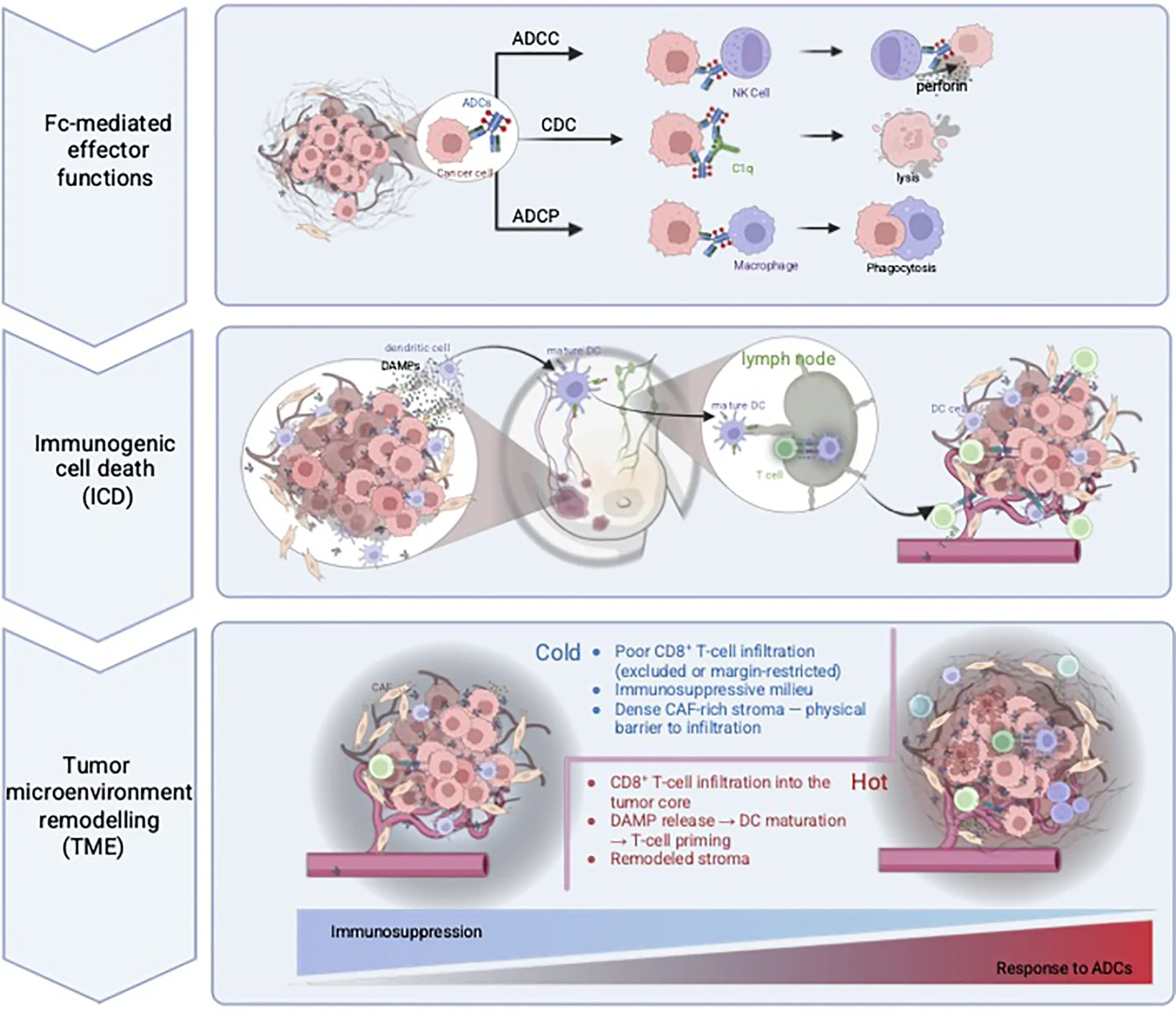
Immunomodulatory mechanisms of ADCs. Schematic representation of the immune activation driven by ADCs. The mechanism comprises Fc-mediated effector functions (ADCC, CDC, and ADCP), the induction of immunogenic cell death (ICD), and the phenotypic conversion of the tumor microenvironment from "cold" to "hot".

#### 3.1.1 Fc-mediated effector functions

ADCs activate the immune system through their Fc region, complementing direct payload-mediated cytotoxicity with antibody-driven immune engagement. The IgG1 Fc region engages immune effectors through three distinct mechanisms: antibody-dependent cell-mediated cytotoxicity (ADCC), complement-dependent cytotoxicity (CDC), and antibody-dependent cellular phagocytosis (ADCP) (17).

In ADCC, the Fab region of the antibody binds to tumor antigens while the Fc region engages CD16 (FcγRIIIa) receptors on natural killer (NK) cells, creating a bridge between the tumor cell and the effector cell (8, 26). This interaction induces NK cell activation and release of cytotoxic granules containing perforin and granzymes, leading to tumor cell lysis. Junttila et al. showed that T-DM1 maintains the ADCC activity of trastuzumab (27), and Ogitani et al. demonstrated that T-DXd maintains ADCC activity with 48.6% maximum cytotoxicity (EC50 3.8 ng/mL) against HER2-positive cells, comparable to the unconjugated anti-HER2 antibody (21). However, direct clinical evidence linking NK cell function to therapeutic response comes from studies with unconjugated HER2-targeting antibodies (28), and the clinical relevance of ADCC for ADCs specifically requires further investigation.

CDC represents a potential mechanism whereby ADCs activate the complement pathway to eliminate tumor cells (27, 29). This mechanism depends on the accumulation of ADCs on the tumor cell membrane via the HER2 receptor. Fc regions recruit C1q, initiating the complement cascade. The process culminates in the assembly of the membrane attack complex (MAC), which forms pores in the tumor cell membrane and causes cell lysis (30). Several studies with unconjugated trastuzumab and pertuzumab showed that high C1q expression was positively associated with improved survival outcomes in HER2-positive breast cancer, while expression of the complement regulatory proteins CD55 and CD59 inversely correlated with clinical outcomes (30, 31). Whether these associations apply to ADC-treated patients remains unexplored.

ADCP represents a further mechanism whereby tumor-associated macrophages (TAMs) contribute to ADC-mediated tumor elimination through Fc-driven phagocytosis. The Fc region of the antibody binds to Fcγ receptors (FcγRs) on macrophages, primarily FcγRIIa (CD32a) and FcγRI (CD64) (32). This binding launches a signaling cascade: FcγRs cluster together and activate Syk kinase, which in turn drives downstream cytoskeletal reorganization. The macrophage membrane extends around the tumor cell followed by scission from the plasma membrane to form a phagosome within the phagocyte (33). Tsao et al. demonstrated that both T-DXd and T-DM1 promote macrophage phagocytosis of HER2-positive breast cancer cells, with efficacy comparable to trastuzumab, indicating that payload conjugation does not impair the inherent Fc-mediated effector functions of the parental antibody (34).

#### 3.1.2 Immunogenic cell death

ICD represents a dynamic multiphase process within the tumor microenvironment in response to therapeutic treatment. Tumor cells exposed to ADCs, before death, enter a stressed state that releases immune signals (DAMPs). Stressed cells expose calreticulin as an “eat me” signal and secrete ATP, while dying cells release HMGB1 from the nucleus. Dendritic cells (DCs) detect these combined signals, and present tumor antigens to T cells in lymph nodes, initiating adaptive immunity (35–37). Preclinical studies reported that ADC cytotoxic payloads can directly modulate dendritic cell function. Martin et al. showed that the precursor of DM1 induces phenotypic and functional DC maturation, promoting antigen uptake by immature DCs (38). Müller et al. reported that different tubulin-targeting cytotoxic agents stimulate DC activation, leading to enhanced CD8+ T cell infiltration into tumors (39).

Gedik et al. demonstrated that T-DM1 induces ICD in HER2-positive breast cancer cells. Once the cytotoxic agent enters the tumor cell, it activates the ICD pathway and triggers secretion of pro-inflammatory signals. In addition, they demonstrated the clinical relevance of ICD in T-DM1 treatment in 57 HER2-positive breast cancer patients. Notably, responding patients showed significantly higher CD8+ T cell infiltration in post-treatment tumor samples compared to non-responders (75% vs 43% CD8-high) (40). T-DXd also induces ICD. Tsao et al. showed that the deruxtecan payload induced higher release of ATP, HMGB1, and calreticulin compared to the DM1 payload, activating DCs through TLR4 and STING pathways. In preclinical models, this promoted stronger CD8+ T cell responses against the tumor (34).

#### 3.1.3 Tumor microenvironment remodeling

The tumor microenvironment (TME) is a complex ecosystem comprising tumor cells, immune cells, stromal components, and extracellular matrix (25, 41). Several studies have reported that the composition of the breast cancer TME influences both disease progression and therapeutic efficacy. Based on immune cell infiltration, the TME can be classified into two main categories: “immune hot” and “immune cold” (41, 42). “Immune hot” tumors are characterized by a Dendritic cells detect these combined signal high concentration of CD8+ cytotoxic T lymphocytes both in the core and periphery of the tumor mass, with high PD-L1 expression on cancer cells, and elevated levels of proinflammatory cytokines such as IFN-γ, which is often, though not invariably, accompanied by high PD-L1 expression on cancer cells (42, 43). “Immune cold” tumors present three different phenotypes. The immune-desert is characterized by the absence of T cells in both the tumor core and margins, while in the immune-excluded phenotype T cells accumulate in the stromal margins but cannot penetrate the tumor parenchyma. Finally, in the immune-suppressed phenotype, T cells are present but functionally exhausted, inhibited by regulatory T cells (Tregs), M2-polarized macrophages, and myeloid-derived suppressor cells (MDSCs) (43).

In breast cancer specifically, this spatial dimension has been characterized directly. Gruosso et al., integrating spatial CD8+ T-cell mapping with laser-capture-microdissection expression profiling in triple-negative breast cancer (TNBC), defined distinct immune microenvironments in which high PD-L1 expression marked both an immunoreactive subtype with good outcome and a poor-outcome immunomodulatory subtype with stromal restriction of CD8+ T cells and stromal PD-L1, the two being distinguished by CD8+ T-cell localization rather than by PD-L1 itself (44). Consistently, Hammerl et al., in 681 TNBC patients, showed that excluded, ignored and inflamed spatial phenotypes, captured by a gene-expression classifier, predict response to anti-PD1 therapy in metastatic TNBC (45). ADCs can modulate the TME toward a more immune-infiltrated phenotype through multiple mechanisms. Preclinical studies have demonstrated that tubulin-targeting payloads significantly reduce intratumoral Tregs, with the most pronounced reduction observed when ADCs are combined with immune checkpoint inhibitors (39). Iwata et al. reported that T-DXd treatment upregulates PD-L1 and MHC class I expression on tumor cells (46).

Clinical studies have provided evidence of these pharmacodynamic immunomodulatory effects. Müller et al. analyzed tumor samples from 28 patients with HER2-positive/HR-positive early breast cancer enrolled in the WSG-ADAPT trial, demonstrating that short-term T-DM1 treatment (3 weeks) significantly increased the percentage of stromal tumor-infiltrating lymphocytes (sTIL: 13.77% → 20.84%, p=0.005) compared to baseline (47). Similarly, SOLTI-1805 TOT-HER3 trial evaluated the biological effects of a single dose of patritumab deruxtecan (HER3-DXd) in 77 patients with HR+/HER2-negative early breast cancer. This study reported that after treatment, there was a significant increase in the CelTIL score, a composite metric incorporating both tumor cellularity and tumor-infiltrating lymphocytes, (median +3.5; p=0.003). Furthermore, the authors observed a trend toward a greater increase in the CelTIL score among responders compared to non-responders (mean difference, +11.9 vs +1.9) (48). Gene expression analysis showed upregulation of immune-related genes (PD-1, CD8, CD19) and suppression of proliferation genes. While these short-term increases in TILs and immune-related gene expression reflect significant pharmacodynamic alterations induced by ADCs, they do not necessarily demonstrate a durable conversion of the tumor immune state (48). Similarly, in the NeoSTAR trial, higher TILs and Ki-67 were reported as predictive of pathologic complete response to neoadjuvant SG in early TNBC (49). Taken together, these clinical studies support a positive correlation between immune infiltrate and ADC response. However, other clinical studies have reported divergent findings. The KATHERINE trial biomarker analysis found that T-DM1 efficacy in HER2-positive early breast cancer was largely independent of CD8 and PD-L1 gene expression in residual disease (50). Similarly, the EMILIA trial, TIL substudy reported no association between stromal tumor-infiltrating lymphocytes and T-DM1 outcome in the advanced HER2-positive setting (51). Larger prospective studies with harmonized methodology are needed to clarify these discrepant findings and to determine whether the predictive value of immune infiltrate depends on the specific ADC, the molecular subtype, or the clinical setting.

### 3.2 Pharmacokinetics

ADC pharmacokinetics requires the quantification of distinct analytical species in circulation: intact ADC, conjugated antibody, total antibody, and free payload. All clinically approved breast cancer ADCs are administered intravenously. The antibody component governs systemic distribution, where the neonatal Fc receptor, FcRn, protects ADCs from lysosomal degradation through an endosomal salvage pathway. Specifically, following non-specific pinocytosis into endothelial and immune cells, the acidic environment of the endosome (pH ~6.0) promotes the binding of the ADC’s Fc region to FcRn. This complex is then recycled back to the cell membrane, where the physiological pH of the bloodstream (pH 7.4) triggers the release of the intact ADC back into circulation, thereby prolonging its half-life (52).

In the bloodstream, ADCs undergo progressive deconjugation over time, leading to dynamic changes in the drug-to-antibody ratio (DAR) (53). Furthermore, as ADCs exit the circulation to bind specific target receptors on cancer cells, they undergo target-mediated drug disposition (TMDD) a saturable pathway that actively contributes to ADC clearance and distribution. Due to their large molecular size, ADCs generally exhibit limited tissue penetration; additionally, non-specific cellular uptake (e.g., via Fc-gamma receptor-mediated endocytosis in immune cells) further contributes to systemic clearance.

Finally, the metabolism of ADCs involves distinct, parallel pathways: the antibody portion undergoes tissue catabolism via lysosomal proteolysis following endocytosis, whereas the released cytotoxic payload is subjected to its own specific metabolic processing (e.g., hepatic CYP450 pathways) prior to excretion (54).

## 4 The evolution of ADCs in breast cancer

As previously mentioned, ADCs are composed of three main components: the antibody, the cytotoxic payload, and the linker. Regarding breast cancer, clinical trials have evaluated ADCs characterized by distinct structural and pharmacological features, reflecting the technological evolution of these compounds.

Early ADC designs often utilized chimeric antibodies, which possessed a high immunogenic potential leading to associated tolerability issues, coupled with unstable linkers at the plasma level and highly variable DARs. To overcome immunogenicity, subsequent constructs transitioned to humanized or fully human antibodies. For example, Damelin et al. conducted a preclinical study on the use of PF-06647263, an ADC composed of a humanized antibody recognizing Ephrin-A4, a GPI-anchored ligand overexpressed in TNBC and ovarian cancer, linked by a cleavable linker (AcBut) to calicheamicin-γ1, a member of the enediyne class of antitumor antibiotics, with a DAR of 4.6. The development of PF-06647263 was discontinued after completion of the phase I study (NCT02078752) in 2018 (55).

Other early constructs presented critical issues as the drug was unable to effectively accumulate inside the tumor cell for mainly 3 reasons: poor drug penetration, problems with cellular uptake, and finally the use of ABC transporters by tumor cells. Therefore, it was decided to increase the potency of the payloads to be effective at low concentrations (56).

Another initial strategy to minimize systemic toxicity while maintaining efficacy involved the development of ADCs with highly stable, non-cleavable linkers and payloads exhibiting low bystander activity. In these constructs, payload release relies exclusively on the complete degradation of the antibody within the tumor cell (57). T-DM1 is composed of an antibody (trastuzumab) that selectively recognizes the HER2 receptor, linked through the MCC linker (non-cleavable) to the payload, DM1 (a maytansine derivative), a potent inhibitor of tubulin polymerization. This ADC has a DAR of ~3.5. Furthermore, the half-life is approximately 4 days, and the clearance is 7.0–13 mL/day/kg (13).

The EMILIA trial, a phase III randomized (n=991), compared T-DM1 with the control regimen of capecitabine plus lapatinib in patients with HER2-positive locally advanced or metastatic breast cancer, previously treated with trastuzumab and taxane. The results demonstrated the superiority of T-DM1 in terms of efficacy. T-DM1 improved PFS (9.6 vs 6.4 months) and overall survival (29.9 vs 25.9 months; HR 0.75, 95% CI 0.64–0.88), with fewer grade ≥3 adverse events than control (48% vs 60%) (58). This led to FDA approval of T-DM1 for second-line systemic treatment of HER2-positive metastatic breast cancer. The KATHERINE trial, a phase III randomized (n=1486), showed that adjuvant T-DM1 improved invasive disease-free survival compared with trastuzumab in patients with residual invasive disease after neoadjuvant therapy (3-year iDFS 88.3% vs 77.0%; HR 0.50, 95% CI 0.39–0.64; p<0.001), with more adverse events in the T-DM1 arm (59).

The DESTINY-Breast03 trial, a phase III randomized (n=524), demonstrated that T-DXd markedly improved PFS (29.0 vs 7.2 months; HR 0.30, 95% CI 0.24–0.38) and overall survival (52.6 vs 42.7 months; HR 0.73, 95% CI 0.56–0.94) in HER2-positive metastatic breast cancer. Grade ≥3 treatment-emergent adverse events occurred in 58.0% vs 52.1%; interstitial lung disease/pneumonitis was a notable T-DXd-specific toxicity (60). For this reason, T-DXd has expanded treatment options beyond HER2-positive disease. The DESTINY-Breast04 Trial confirmed its efficacy in the HER2-low breast cancer setting with FDA approval for that indication in 2022 (61). Indeed, this drug utilizes a GGFG tetrapeptide linker (maleimide glycine-glycine-phenylalanine-glycine) that is enzymatically cleavable. This tetrapeptide is degraded by lysosomal enzymes such as cathepsins B and L, which are highly expressed in tumor cells. This allows for a high DAR of 7.7, which is approximately the theoretical maximum for conventional cysteine conjugation, while showing excellent stability. This characteristic may explain how it delivers enough payload even to tumors with low HER2 expression (56).

Expanding the pharmacological landscape beyond HER2, recent platforms utilize novel cleavable linker chemistries, such as pH-sensitive linkers containing a PEG (polyethylene glycol) segment to enhance water solubility. This structural design is exemplified by SG, which utilizes a CL2A linker to conjugate the SN-38 payload to a humanized anti-TROP2 antibody, achieving a high average DAR of 7.6. This configuration enables a highly effective mechanism of action. First, the ADC binds to TROP2 on the tumor cell and is internalized; the payload then undergoes intracellular trafficking and enters the lysosomes, where it is released following antibody catabolism and linker hydrolysis to induce apoptosis. Furthermore, this ADC exerts a potent bystander effect due to the release of SN-38 from the target cell or directly within the extracellular space, facilitated by the pH-sensitive carbonate bond of the linker. In this manner, neighboring cancer cells are affected independently of TROP2 expression, contributing to an amplified anti-tumor effect that may limit the development of resistance (22).

The ASCENT trial, a phase III randomized (SG n=235 vs chemotherapy n=233), enrolled patients with metastatic TNBC previously treated with taxanes. SG significantly improved PFS (5.6 vs 1.7 months; HR 0.41, 95% CI 0.32–0.52; p<0.001) and OS (12.1 vs 6.7 months; HR 0.48, 95% CI 0.38–0.59; p<0.001), with an ORR of 35% vs 5%; grade ≥3 neutropenia occurred in 51% vs 33% (62).

Another significant advancement in TROP2-targeted therapy is represented by datopotamab deruxtecan (Dato-DXd), which employs a different technological approach compared to SG. Indeed, Dato-DXd utilizes a tetrapeptide-based cleavable linker with DAR of 4. This linker is designed to be cleaved by lysosomal enzymes such as cathepsins after internalization into tumor cells. This strategy was selected to maximize the therapeutic window (63). TROPION-Breast01 trial, a phase III randomized (n=732), showed that Dato-DXd significantly improved PFS compared to chemotherapy in patients with HR+/HER2- metastatic breast cancer (6.9 vs 4.9 months; HR 0.63, 95% CI 0.52–0.76; p<0.0001). In addition, it demonstrated a favorable safety profile with grade ≥3 treatment-related adverse events occurring in 21% vs 45% of patients (64). Dato-DXd was approved by the FDA in January 2025 for previously treated HR+/HER2− metastatic breast cancer. More recently, TROPION-Breast02, Dato-DXd was approved in May 2026 for first-line metastatic TNBC in patients not candidates for PD-1/PD-L1 inhibitor therapy.

Another approach is represented by the development of MEDI4276, a biparatopic antibody targeting two distinct epitopes on HER2, with the payload attached by site-specific conjugation, yielding an average DAR of 4 (56). Unfortunately, during the phase I study, this drug demonstrated intolerable adverse events including hepatotoxicity (grade 3/4 AST/ALT elevations), severe gastrointestinal toxicity, and peripheral neuropathy, leading to its discontinuation despite evidence of clinical activity (65).

## 5 Resistance mechanisms

Resistance to chemotherapeutic and molecularly targeted agents shares common features: alterations in the drug target, activation of prosurvival pathways, and ineffective induction of cell death (66). In the context of antibody-drug conjugates, resistance has recently been summarized as encompassing target antigen alterations, cell-intrinsic mechanisms that overcome payload cytotoxicity, and the pro-tumorigenic tumor microenvironment (67).

The principal mechanisms of resistance to ADCs in breast cancer are summarized in **Supplementary Table 1**. While clinical failure can manifest as intrinsic (primary) or acquired resistance and can be driven by either agent-specific alteration (e.g., MUC4 masking) or payload-class cross-resistance (e.g., TOP1 mutations), we have organized these multimodal mechanisms along our two defining axes: molecular and immunological. Furthermore, within these axes, we aim to delineate causal mechanistic factors from treatment-response biomarkers (such as immune infiltration metrics).

In addition, as illustrated in **Figure 2**, mechanisms of resistance can be multimodal and multistep.

**Figure 2:**
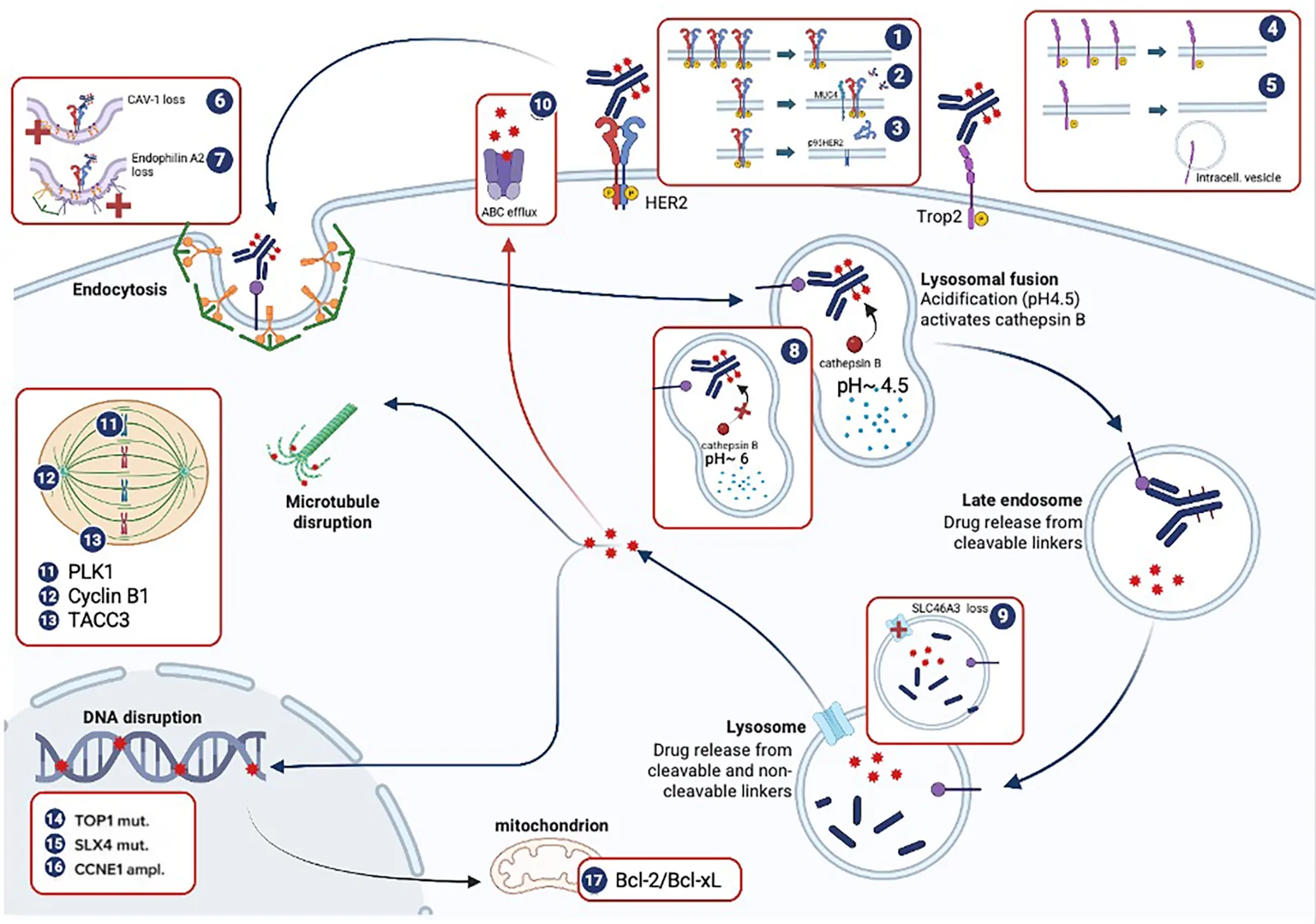
Molecular and cellular mechanisms of resistance to antibody-drug conjugates (ADCs). Schematic overview of the multi-step ADC intracellular lifecycle and the corresponding alterations leading to therapeutic evasion. (1) Decrease in target antigen expression levels on the cell surface. (2) Steric masking by membrane-bound proteins (e.g., MUC4) blocking ADC binding. (3) Expression of truncated antigen isoforms (e.g., p95HER2) lacking the extracellular binding domain. (4) Downregulation or loss of alternative target antigens (e.g., Trop2). (5) Defective target antigen internalization or altered intracellular vesicle trafficking. (6) Loss of caveolin-1 (CAV-1), impairing caveolae-mediated endocytosis. (7) Loss of Endophilin A2, disrupting clathrin-independent endocytic pathways. (8) Defective lysosomal acidification ( instead of ) impairing cathepsin B activation and linker cleavage. (9) Loss of the lysosomal transporter SLC46A3, preventing payload escape into the cytoplasm. (10) Upregulation of ATP-binding cassette (ABC) efflux pumps actively extruding the payload. (11) Overexpression of PLK1 involved in mitotic progression. (12) Failure to accumulate cyclin B1 upon ADC treatment, preventing mitotic arrest and subsequent apoptotic response. (13) Alterations in TACC3, contributing to spindle assembly disruptions. (14) Somatic mutations in DNA Topoisomerase I that confer cross-resistance to both SN-38 and deruxtecan payloads. (15) Alterations or loss of the SLX4 DNA-repair scaffold. (16) Amplification or overexpression of cyclin E1. (17) Upregulation of anti-apoptotic proteins Bcl-2 and Bcl-xL.

### 5.1 Receptor-related resistance

#### 5.1.1 HER2 downregulation and intratumoral heterogeneity

Mosele et al., during the DAISY trial, investigated a subset of 20 patients with paired tumor biopsies obtained at baseline and at resistance to T-DXd, comparing HER2 expression by immunohistochemistry. They reported that 13 of 20 patients (65%, 95% CI 40.8–84.6) showed decreased HER2 expression at resistance, with shifts to lower IHC categories (e.g., from IHC 3+ to IHC 2+ or IHC 1+, and from IHC 1+ to IHC 0) (68).

However, it is crucial to note that spatial and quantitative antigen modulations (such as target downregulation, shedding, or MUC4 masking) have been predominantly characterized in HER2-amplified models. The clinical emergence of HER2-low and HER2-ultralow breast cancers as distinct therapeutic entities has recently been underscored by the DESTINY-Breast06 trial, which demonstrated T-DXd efficacy even in tumors with HER2 IHC 0 and faint, incomplete membrane staining (i.e., HER2-ultralow) (99). This calls for a re-evaluation of the paradigm of acquired resistance. When target density is already at the minimal biological threshold, further antigen loss likely exerts a diminished proportional impact on resistance. Instead, in these low-expression profiles, the clinical efficacy of highly cleavable ADCs relies heavily on a robust bystander effect to compensate for sparse direct target engagement. Consequently, we hypothesize that in HER2-low and ultralow tumors, acquired resistance is predominantly driven by intracellular, payload-centric mechanisms, such as the upregulation of efflux transporters or altered apoptotic pathways, rather than further target modulation.

#### 5.1.2 MUC4 masking

MUC4 is a transmembrane glycoprotein that interacts with the extracellular domain of HER2, thereby inhibiting the binding between trastuzumab and its receptor. Mercogliano et al. demonstrated that TNFα induces MUC4 upregulation through NF-κB pathway activation, conferring resistance to both trastuzumab and T-DM1. *In vitro*, TNFα-overexpressing HER2-positive cells exhibited 10-fold reduced sensitivity to T-DM1; MUC4 silencing sensitized cells to T-DM1. In addition, in xenograft models, TNFα blockade downregulated MUC4 and resensitized tumors to trastuzumab. Finally, clinical analysis of 78 HER2-positive patients confirmed MUC4 as an independent predictor of poor disease-free survival (HR 5.43, p=0.008) (74). A preclinical study demonstrated that co-administration of trastuzumab with a TNFα inhibitor induced MUC4 downregulation and successfully overcame trastuzumab resistance (75).

#### 5.1.3 p95HER2 shedding

HER2 is proteolytically cleaved (shedding) by ADAM10, generating two fragments: a truncated membrane-bound form, p95HER2, and a soluble extracellular domain (HER2-ECD) released into the circulation. Since trastuzumab recognizes HER2-ECD, shedding directly impairs antibody binding and represents a mechanism of resistance to trastuzumab-based therapy. Yang et al. demonstrated *in vitro* and in mouse models that DPAGT1, which stabilizes ADAM10, is required for HER2 shedding, and that pharmacological inhibition of DPAGT1 overcame trastuzumab resistance in HER2-positive breast cancer (100).

#### 5.1.4 TROP2 alterations

Wilson et al., in preliminary data, demonstrated that an N-terminally truncated form of TROP2 is a mechanism of resistance to ADCs *in vitro*. Their study reported that triple-negative breast cancer cells expressing the truncated TROP2 showed reduced binding and reduced cytotoxicity to both SG and Dato-DXd (101). Coates et al. reported a different receptor alteration in a patient with metastatic TNBC progressing on SG: a missense TACSTD2/TROP2 T256R mutation, which conferred resistance through defective plasma membrane localization and reduced surface binding by the SG antibody (77). These two independent mechanisms, protein truncation and somatic mutation, both alter TROP2 surface availability and reduce ADC efficacy.

### 5.2 Internalization and trafficking defects

T-DM1 activity requires internalization of the HER2-antibody complex from the cell surface and trafficking to the lysosome, where the non-cleavable linker is degraded and the DM1 payload released (79, 102).

#### 5.2.1 Lipid raft/caveolin-1 alteration

HER2 receptors on tumor cell membranes localize within cholesterol-rich microdomains called lipid rafts, where caveolin-1 (CAV-1) mediates internalization of the HER2-antibody complex. Liang et al. demonstrated that lipid raft inhibitors block T-DM1 degradation, while clathrin inhibition does not, indicating a predominant role for caveolae-mediated rather than clathrin-mediated endocytosis in T-DM1 trafficking (102).

#### 5.2.2 Endophilin A2 deficiency

Endophilin A2 (Endo II), encoded by the SH3GL1 gene, is an endocytic adaptor protein involved in clathrin-independent endocytosis. Baldassarre et al. demonstrated that in HER2-positive breast cancer cell lines, silencing of Endo II significantly reduced the *in vitro* cytotoxicity of both trastuzumab and T-DM1, indicating that Endo II-mediated internalization of the HER2-antibody complex is required for the activity of T-DM1 (78).

### 5.3 Lysosomal dysfunction

#### 5.3.1 SLC46A3 loss

Kinneer et al. discovered that resistance to T-DM1 and other ADCs with non-cleavable maytansinoid and selected PBD payloads is driven by the loss of SLC46A3. Indeed, when T-DM1 enters the tumor cell, it is degraded in lysosomes releasing the catabolite Lys-MCC-DM1. This catabolite, being positively charged, cannot spontaneously cross the lysosomal membrane. SLC46A3 functions as a pump that actively transports the drug out of the lysosome (83). Without SLC46A3, the drug remains trapped in the lysosome. Consequently, cells without SLC46A3 are resistant to T-DM1.

#### 5.3.2 Lysosomal alkalinization and reduced cathepsin B activity

Ríos-Luci et al. have shown that lysosomal dysfunction is a resistance mechanism for T-DM1 in HER2-positive breast cancer. Their *in vitro* studies reportedthat resistant cells exhibit increased lysosomal pH and a 40-50% reduction in cathepsin B activity, which impairs proteolytic drug processing. Furthermore, their preclinical studies confirmed that tumors from resistant cells paradoxically accumulated more T-DM1, demonstrating that the drug enters but remains trapped. This mechanism was further confirmed in patient-derived xenograft (PDX) models from T-DM1-resistant HER2-positive samples, establishing lysosomal pH alteration as a candidate mechani**s**m, whose clinical relevance remains to be established (84).

### 5.4 Payload efflux mechanisms

The upregulation of ATP-binding cassette (ABC) transporters presents a secondary barrier by mediating the active efflux of the payload across the plasma membrane. Recent comparative analyses demonstrate that higher expression of these multidrug resistance (MDR) pumps significantly increases the IC50 of DXd relative to exatecan, highlighting a differential susceptibility to cellular extrusion between specific payload derivatives. Notably, pharmacological inhibition of ABC transporters can sensitize resistant cells to DXd- and SN-38-based conjugates, offering a promising strategy to bypass this defensive mechanism (103).

#### 5.4.1 MDR1/ABCB1 upregulation

Li et al. discovered that upregulation of MDR1, a transmembrane protein responsible for expelling the cytotoxic drug (DM1) from the cell, was a *in vitro* resistance mechanism. This mechanism was evaluated in a preclinical study, which demonstrated that pharmacological inhibition of MDR1 could reverse T-DM1 resistance in cell models. While these findings provide a rationale for combining T-DM1 with MDR1 inhibitors as a potential therapeutic strategy, the clinical utility of this approach requires further validation (86).

#### 5.4.2 ABCC1 upregulation

Sledge et al. analyzed a real-world cohort of 2799 patients with breast cancer treated with T-DXd and showed that higher ABCC1 expression in pre-treatment samples was associated with shorter T-DXd-specific OS, independently of HER2 status (85). The combination of high HER2 and low ABCC1 identified the best outcomes, supporting ABCC1-mediated payload efflux as a resistance mechanism (85, 104).

### 5.5 Payload activity resistance

Besides efflux transporter upregulation, payload resistance can be achieved by stopping payload activity through modification of intracellular protein targets.

#### 5.5.1 TOP1 mutations

Abelman et al. reported that in a cohort of 31 patients with metastatic breast cancer treated with ADCs, 4 (12.9%) developed somatic TOP1 mutations at disease progression, compared to only 0.7% in patients not treated with ADCs. These TOP1 mutations conferred resistance to both SN-38 and deruxtecan payloads, with clinical cross-resistance evidenced by shorter treatment duration on subsequent ADCs (88).

#### 5.5.2 Defective cyclin B1 induction

Sabbaghi et al. demonstrated that three independent T-DM1-resistant clones derived from HER2-positive breast cancer cell lines failed to accumulate cyclin B1 upon T-DM1 treatment, while ectopic cyclin B1 induction partially restored sensitivity. The same defect was observed ex vivo in fresh tumor explants from 18 HER2-positive breast cancer patients, where cyclin B1 induction after T-DM1 paralleled the apoptotic response (87).

#### 5.5.3 PLK1 overexpression

In a related mechanism, Saatci et al. identified PLK1 overexpression as a driver of T-DM1 resistance. High PLK1 activity in HER2-positive breast cancer cells maintains mitotic progression and bypasses spindle assembly checkpoint (SAC) activation, preventing T-DM1-induced cell death. Genetic and pharmacological inhibition of PLK1 restored sensitivity in both acquired and *de novo* T-DM1-resistant HER2-positive cell line models, including 3D culture and xenograft validation (89).

### 5.6 Replication stress and DNA repair alterations

Genetic control of replication stress and DNA repair shapes the response to ADCs, and its disruption can confer resistance to this drug class. Two genomic determinants have emerged in this context: CCNE1 amplification and SLX4 loss.

#### 5.6.1 CCNE1 amplification

CCNE1 encodes cyclin E1, which complexes with CDK2 to drive the G1/S transition; its amplification or overexpression allows tumor cells to bypass CDK4/6-mediated cell-cycle arrest and sustains replication stress. Yamaguchi et al., in 114 patients with HER2-positive advanced gastric cancer treated with T-DXd, found CCNE1 amplification in 36% of tumors and an association with shorter PFS compared with non-amplified tumors (131 vs 189 days; HR 1.90; p= 0.044) (105). A functional link was established preclinically by DiPeri et al.: in HER2-positive models, cyclin E overexpression reduced sensitivity to T-DXd while CCNE1 knockdown restored it, the DXd payload itself induced cyclin E expression, and the Wee1 inhibitor adavosertib was synergistic with T-DXd in CCNE1-amplified patient-derived xenografts (90). Whether CCNE1 amplification predicts ADC resistance in breast cancer specifically remains unexplored.

#### 5.6.2 SLX4 loss

SLX4 is a DNA-repair scaffold that coordinates structure-specific endonucleases to resolve replication-associated recombination intermediates (106). In the DAISY trial, SLX4 mutations were enriched in biopsies obtained at resistance to T-DXd (3/21, 14%) compared with pretreatment samples (3/89, 3%) and TCGA-BRCA (1.5%); *in vitro*, SLX4 knockdown in HER2-positive cell lines reduced sensitivity to the DXd payload (68). These data nominate SLX4 as a candidate determinant of ADC resistance, although its low frequency limits current clinical inference.

### 5.7 Apoptosis resistance

A fundamental hallmark of cancer is the evasion of programmed cell death, which frequently drives resistance to cytotoxic therapies, including those delivered by ADCs. Within this framework, the Bcl-2 protein family serves as a critical checkpoint governing cell survival and apoptotic thresholds (107).

In breast cancer, particularly in HER2-positive malignancies, an elevated Bcl-2/Bax ratio has been closely associated with an anti-apoptotic phenotype and resistance to targeted agents. Preclinical models of acquired trastuzumab resistance demonstrate that upregulated Bcl-2-mediated survival signaling, often maintained via compensatory PI3K and IKK pathway activation, shields tumor cells from anti-HER2 therapies (108). Crucially, this high apoptotic threshold directly compromises the efficacy of ADCs, which ultimately rely on the activation of apoptotic cascades to execute payload-mediated cytotoxicity.

The impact of Bcl-2 on ADCs’ efficacy was evaluated by Zoeller et al. in five HER2-expressing patient-derived breast cancer xenograft models. Pharmacologic inhibition of Bcl-2/XL with navitoclax substantially increased T-DM1’s cytotoxic activity in multiple models and notably, combined therapy enhanced therapeutic responses even in tumors with low HER2 expression; in addition, toxicity was managed with intermittent doses (91). These data indicate that Bcl-2/XL inhibition may improve T-DM1 efficacy and potentially broaden therapeutic effect of HER2-directed ADCs beyond conventional HER2-amplified tumors.

### 5.8 Immunological escape mechanisms

The immunological mechanisms are shown in **Figure 3**.

**Figure 3:**
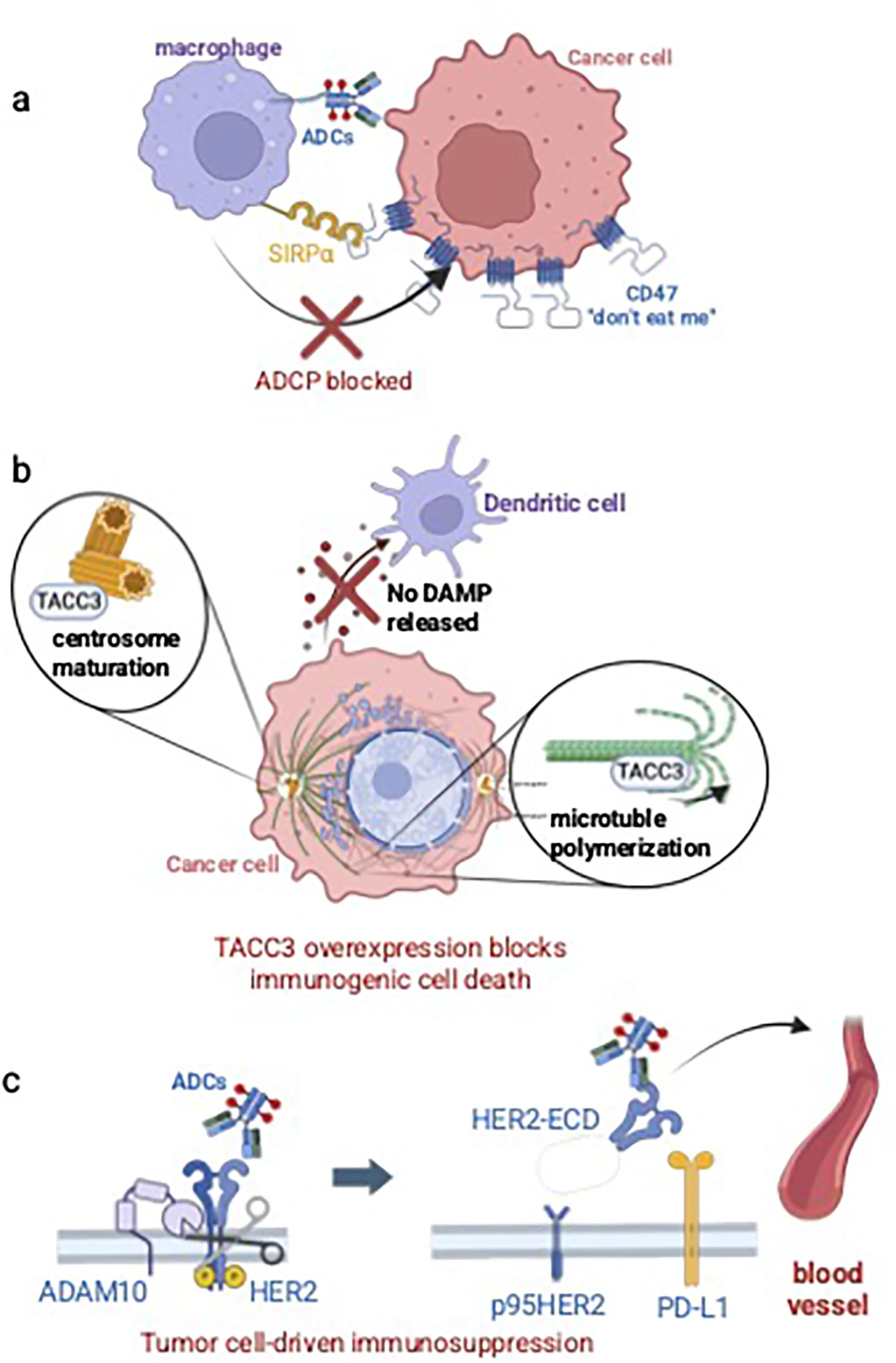
Mechanisms of immunological escape and microenvironmental resistance to ADCs. Schematic overview of the tumor-mediated pathways that impair antibody- and payload-driven immune responses. **(A)** Impaired ADCP via the CD47 -SIRP axis: interaction between the upregulated CD47 "don't eat me" signal on the cancer cell surface and SIRP expressed on macrophages. **(B)** Loss of immunogenic cell death (ICD) via TACC3 overexpression: high levels of TACC3 stabilize the mitotic machinery by promoting centrosome maturation and microtubule polymerization. **(C)** Tumor cell-driven immunosuppression and HER2 shedding: proteolytic cleavage of full-length HER2 by the metalloproteinase ADAM10 leads to the shedding of the extracellular domain (HER2-ECD) into the blood vessel, leaving the truncated p95HER2 isoform and inducing PD-L1 expression on the cell membrane.

#### 5.8.1 Impaired ADCC

The efficacy of ADCC depends on the affinity between the IgG1 Fc region and CD16 (FcγRIIIa) expressed on NK cells. The FCGR3A V158F polymorphism produces two allotypes: V/V homozygotes show the highest binding affinity and the strongest NK-mediated cytotoxic response, whereas F/F homozygotes show reduced affinity and impaired ADCC. In HER2-positive metastatic breast cancer, two retrospective series reported that V/V genotype was associated with better objective response rate and PFS on trastuzumab-based therapy compared to F/F (Musolino et al., 2008, n=54; Abdel-Wahed et al., 2024, n=80) (95, 96). Confirmation in larger adjuvant cohorts has been mixed: NSABP B-31 supported a predictive role of the polymorphism, with F/F homozygotes deriving less benefit from trastuzumab (Gavin et al., 2017, n=1251) (97), whereas in NCCTG N9831 no significant association was observed (Norton et al., 2014, n≈1170) (98). Importantly, the clinical evidence regarding the FCGR3A polymorphism derives exclusively from these trastuzumab-treated cohorts. While T-DM1 and T-DXd retain the trastuzumab IgG1 backbone and preserve ADCC activity, an effect of the FCGR3A genotype on clinical outcomes with ADCs has not been established. Its role as a potential modulator of ADC efficacy remains theoretical.

#### 5.8.2 Impaired ADCP

Tsao et al. identified CD47 upregulation as a mechanism of immunological resistance to T-DXd. The DXd payload increases surface CD47 expression on HER2-positive breast cancer cells, while the DM1 payload decreases it; this effect is intrinsic to the cytotoxic agents and is reproduced by the corresponding ADCs. CD47 acts as a “don’t eat me” signal binding SIRPα on macrophages and blocking ADCP of trastuzumab-opsonized tumor cells. In addition, the authors showed that CD47 deletion directly increased T-DXd-mediated ADCP, confirming CD47 as a causal barrier to the immunological component of T-DXd activity (34). In parallel, Upton et al. showed that the constitutively high CD47 levels of HER2-positive breast cancer cells limit trastuzumab-mediated ADCP independently of payload, providing a complementary baseline mechanism of resistance to HER2-directed antibody therapy (109) (**Figure 3A**).

Mechanistically, CD47 upregulation is a known consequence of DNA damage, occurring through the activity of MRE11, a sensor of DNA double-strand breaks (DSBs) (110). DXd, as a topoisomerase-I inhibitor, is expected to generate DSBs and engage this pathway, while the microtubule-targeting agent DM1 acts in the opposite direction. Whether this immunological resistance mechanism is payload-class specific, and whether it generalizes to other topoisomerase-I-based ADCs (SG, Dato-DXd), remains an open question.

#### 5.8.3 Loss of immunogenic cell death

Gedik et al. identified TACC3 overexpression as a mechanism of immunological resistance to T-DM1. This protein stabilizes the mitotic spindle, enabling bypass of DM1-induced spindle assembly checkpoint (SAC) activation and consequently leading to loss of ICD induction (**Figure 3B**). In a clinical correlative analysis of 57 HER2-positive patients, non-responders exhibited increased TACC3 expression and reduced CD8+ infiltration (75% vs 43%, CD8-high tumors in responders vs non-responders), proposing TACC3 as a candidate biomarker of immune-mediated T-DM1 resistance, although its predictive value requires further validation. Furthermore, the authors conducted preclinical studies demonstrating that TACC3 inhibitors in combination with T-DM1 enhanced therapeutic efficacy and overcame resistance in T-DM1-resistant models (40).

#### 5.8.4 Tumor microenvironment immunosuppression

The composition of the TME influences ADC efficacy beyond the cancer cell itself. In the NeoSTAR phase II trial, Spring et al. reported that in 50 patients with localized TNBC treated with neoadjuvant SG monotherapy, baseline stromal tumor-infiltrating lymphocyte (sTIL) density was significantly predictive of pathological complete response, with median TIL values of 55% in pCR cases versus 31% in non-pCR cases (p=0.002) (49). These findings highlight the prognostic role of immune infiltration under ADC therapy and motivate investigation into the specific TME-related mechanisms that govern resistance. Three distinct axes have emerged: tumor-cell programming of an immunosuppressive niche, extracellular matrix remodeling driving epithelial-mesenchymal transition, and stromal modulation by cancer-associated fibroblasts.

##### 5.8.4.1 Tumor cell-driven immunosuppression

Hu et al. demonstrated that p95HER2 reprograms HER2-positive breast cancer cells into an immunosuppressive state. The truncated receptor induces PD-L1 and IL-6, uniquely activating JAK–STAT signaling, and its expression reduces intratumoral T cells, NK cells and dendritic cells. In addition, the authors showed in preclinical models that, in mice bearing HER2-positive breast tumors treated with T-DXd, tumour growth was inhibited by 52% in the group expressing full-length HER2 alone, whereas no effect was observed in the group co-expressing full-length HER2 and p95HER2. Treatment increased dendritic and T-cell infiltration only in tumors without p95HER2. Finally, the authors compared these findings with data from the FinHer trial, a cohort treated with adjuvant trastuzumab rather than ADCs, in which a higher p95HER2/HER2 ratio correlated with reduced T-cell activation (p=0.0098) (70). However, the mechanism generating p95HER2 in human tumors remains unclear, and different groups have characterized different truncated species under the same name. The route depicted in **Figure 3C** is therefore a working hypothesis requiring further investigation.

##### 5.8.4.2 ECM remodeling and EMT

Chen et al. identified MMP1 (matrix metalloproteinase 1) overexpression as a resistance mechanism to SG in hormone receptor-positive breast cancer, showing in preclinical models that MMP1 levels are approximately 30-fold higher in HR-positive tumor tissues compared to normal breast. Mechanistically, MMP1 has been shown to activate the NF-κB pathway and promote epithelial-mesenchymal transition (EMT), thereby contributing to SG resistance (94).

##### 5.8.4.3 Cancer-associated fibroblasts

Beyond ECM remodeling, cancer-associated fibroblasts (CAFs) themselves can mediate ADC resistance through both physical and stromal-immune mechanisms. Zhang et al. demonstrated in preclinical models (PDX and CDX) that excessive CAFs form a fibrotic barrier that reduces tumor uptake of ADCs, with low-response T-DXd tumors showing significantly higher FAP staining and collagen deposition than high-response tumors. Cancer cells transformed normal fibroblasts into FAK-activated CAFs, and treatment with the FAK inhibitor IN10018 reduced CAF density, increased trastuzumab tumor uptake, and significantly enhanced antitumor efficacy when combined with T-DXd or TROP2-targeted ADCs (92).

Clinical relevance came from the randomized PREDIX HER2 phase II trial of neoadjuvant T-DM1 versus docetaxel/trastuzumab/pertuzumab, where Manikis et al., in a conference report, showed that total CAF density did not predict pathological complete response, but specific subpopulations did. Higher baseline densities of PDGFRα+/FAP- CAFs were associated with residual disease, with the prognostic effect retained specifically in the T-DM1 arm (adjusted p<0.001) and lost in the THP arm. From the immune panel, higher densities of CD20+ B cells and CD4+/FoxP3+ regulatory T cells were associated with pCR (adjusted p=0.012), with the B cell effect remaining significant in the T-DM1 arm (adjusted p=0.015) (93).

## 6 Current strategies to address resistance

Currently, resistance to ADCs represents a major clinical challenge limiting the efficacy of this pharmacological class in breast cancer. As discussed in previous sections, ADCs produce significant immunomodulatory effects. These immunological mechanisms introduce a strong rationale for combining ADCs with other therapeutic approaches to enhance efficacy and overcome resistance.

### 6.1 ADC + immune checkpoint inhibitors

ADCs combine immunogenic cell death induction with reduction of TME immunosuppression, providing a biological rationale for combination with immune checkpoint inhibitors. The KATE2 trial (NCT02924883) was the first randomized, double-blind, phase II study evaluating the addition of atezolizumab to T-DM1 versus T-DM1 plus placebo in 330 patients with HER2-positive advanced or metastatic breast cancer previously treated with trastuzumab and taxane. The authors reported that the trial did not meet its primary PFS endpoint. However, in the PD-L1-positive subgroup (immune cell ≥1%), median PFS was 8.5 vs 4.1 months (HR 0.60; 95% CI 0.32–1.11). In the PD-L1-negative subgroup, median PFS was 6.8 vs 8.2 months (HR 1.02; 95% CI 0.60–1.74), with no evidence of benefit (111). These results demonstrated that the combination therapy appears beneficial only in patients expressing PD-L1, while PD-L1-negative patients showed no advantage from the combination. Based on these findings, the phase III KATE3 trial (NCT04740918) was initiated, enrolling only patients with HER2-positive and PD-L1-positive disease (112).

The BEGONIA trial (NCT03742102), a single arm study, evaluated Dato-DXd plus durvalumab as first-line treatment in metastatic TNBC in 62 patients (arm 7). The authors reported that 87% of the cohort had PD-L1-low tumors, with a confirmed ORR of 79.0%. These results suggest that responses were comparable irrespective of PD-L1 status (79.6% in PD-L1-low vs 71.4% in PD-L1-high tumors) (113, 114). However, the single-arm design precludes definitive conclusions regarding PD-L1 independence.The ASCENT-04/KEYNOTE-D19 phase III trial (NCT05382286) enrolled 443 patients with previously untreated, PD-L1-positive (CPS ≥10) locally advanced or metastatic TNBC, randomized 1:1 to SG plus pembrolizumab versus chemotherapy plus pembrolizumab. SG plus pembrolizumab significantly improved PFS by blinded independent central review (11.2 vs 7.8 months; HR 0.65, 95% CI 0.51–0.84; p<0.001), with a higher ORR (60% vs 53%) and longer duration of response (16.5 vs 9.2 months). Overall survival data were immature at a median follow-up of 14 months (HR 0.89, 95% CI 0.62–1.29). In addition, it demonstrated a favorable safety profile with grade ≥3 treatment-related adverse events occurring in 71% vs 70% of patients (115). In June 2026 the FDA approved SG plus pembrolizumab for the first-line treatment of PD-L1 CPS ≥10 metastatic TNBC. Additional ongoing studies are exploring ADC-ICI combinations in earlier disease settings, including the TRUDI trial (NCT05795101), a phase II neoadjuvant study of T-DXd plus durvalumab in HER2-expressing inflammatory breast cancer (116). These trials differ substantially in subtype (HER2-positive vs TNBC), treatment line, and design, and were not intended for cross-comparison; differences in outcome cannot be attributed to specific biological factors. Further studies are needed to clarify which factors predict response to ADC-ICI combinations.

### 6.2 ADCs + tyrosine kinase inhibitors

Receptor-related mechanisms represent a major cause of ADC resistance by preventing antibody binding and subsequent payload internalization. To overcome these limitations, several studies have explored the combination of ADCs with TKIs based on their ability to modulate HER2 trafficking and surface availability.

HER2CLIMB-02 (NCT03975647) is a randomized, double-blind, placebo-controlled, phase III trial that enrolled 463 patients with HER2-positive locally advanced or metastatic breast cancer. Tucatinib plus T-DM1 improved median PFS compared with T-DM1 alone (9.5 vs 7.4 months; HR 0.76, 95% CI 0.61–0.95; p=0.016), a benefit observed across all prespecified subgroups rather than in patients selected for a defined resistance mechanism. The addition of tucatinib increased toxicity, with more frequent hepatic events (28.6% vs 7.3%) and treatment discontinuations due to adverse events (20.3% vs 11.2%) (117). Despite these encouraging results, several questions remain unanswered. First, the optimal TKI class for ADC combination is unclear. In fact, reversible TKIs (e.g., tucatinib, lapatinib) inhibit HER2 phosphorylation and reduce receptor ubiquitination, resulting in accumulation of inactive HER2 at the cell surface (118), whereas irreversible TKIs (e.g., neratinib, afatinib, pyrotinib) dissociate HSP90 from HER2, triggering receptor ubiquitination and subsequent degradation (119). Second, whether these benefits extend to other ADCs remains to be determined. Ongoing trials including HER2CLIMB-04 (NCT04539938) evaluating T-DXd plus tucatinib will help address these gaps.

### 6.3 ADC + PARP inhibitors

Cardillo et al. investigated the combination of SG with PARP inhibitors *in vitro* and in preclinical TNBC models. The rationale was based on maximization of DNA damage: SN-38 generates double-strand breaks during replication, while PARPi blocks their repair. The combination showed synergistic activity in both BRCA-mutated and BRCA1/2 wild-type cell lines, with improved antitumor efficacy in xenograft models and no significant hematologic toxicity in mice (120).

The phase Ib SEASTAR study (NCT03992131) evaluated rucaparib combined with SG in six patients with advanced solid tumors (TNBC, ovarian, and urothelial cancer), with or without HRR gene mutations. The combination showed clinical activity, with 3/6 patients achieving a confirmed partial response, but was associated with significant hematologic toxicity (grade 3/4 neutropenia in all patients), requiring dose modifications and growth factor support. The authors concluded that further evaluation was warranted but the recommended phase II dose was not formally established (121).

Bardia et al. hypothesized that the toxicity of concurrent dosing was due to simultaneous systemic exposure: residual circulating SN-38 combined with PARPi causes severe myelosuppression. They proposed sequential administration as a viable strategy to mitigate toxicity. To evaluate this approach, they conducted a phase Ib trial (NCT04039230) enrolling 30 patients with metastatic TNBC. The concurrent cohort confirmed unacceptable toxicity, while the sequential cohort, receiving SG on days 1 and 8 followed by talazoparib on days 15–21, reported no dose-limiting toxicities, a median PFS of 7.6 months, and an ORR of 42%. These preliminary findings support the clinical feasibility of this sequential schedule and suggest that ADC delivery can be leveraged to create pharmacological windows enabling previously unfeasible combinations (122).

### 6.4 ADC + CDK4/6i

Spring et al. conducted a phase Ib dose-escalation trial of ribociclib combined with T-DM1 in 12 patients with HER2-positive metastatic breast cancer, primarily to assess safety and dose/schedule feasibility. The original concurrent dosing schedule (ribociclib on days 5–18 of a 21-day cycle) was modified to days 8–21 due to overlapping myelosuppression, allowing sequential rather than concurrent drug exposure. With the revised schedule, ribociclib 400 mg was determined as the recommended phase II dose. While the study was not powered to formally evaluate efficacy, preliminary activity was observed, with a median PFS of 10.4 months (95% CI: 2.7–19.3), reflecting the wide statistical uncertainty expected in such a small cohort. The most common grade 3 toxicities were neutropenia (33%), leukopenia (33%), and anemia (25%) (123). The pharmacological rationale for sequential dosing is twofold. First, Salvador-Barbero et al. demonstrated that CDK4/6 inhibitors arrest cells in G0/G1, antagonizing the effect of mitosis-targeting agents (including taxanes and DM1) when administered concurrently; conversely, sequential administration of CDK4/6 inhibitors after antimitotic agents potentiates antitumor activity by impairing DNA repair (124). Second, T-DM1-induced thrombocytopenia is primarily mediated by DM1-induced impairment of megakaryocyte differentiation, while CDK4/6 inhibitors are known to cause neutropenia and leukopenia as their main hematologic toxicities: the two agents converge on bone marrow progenitor compartments through distinct mechanisms, making sequential dosing pharmacologically rational (125).

## 7 Conclusions and considerations

A century after Paul Ehrlich’s “magic bullet”, antibody-drug conjugates represent the closest realization of that concept in breast cancer. After a decade of clinical use of T-DM1 and the widespread use of T-DXd since its initial approval in 2019, resistance has emerged as the rule rather than the exception (67). Resistance does not result from a single mechanism, but from the convergence of interconnected biological domains: a molecular one, driven by tumor genomic and trafficking alterations, and an immunological one, mediated by the tumor microenvironment (67, 69, 74).

Integrated molecular and immunopathological profiling can address this complexity. With NGS now routinely available alongside immunohistochemistry, the clinical question may shift from which drug a patient is eligible for to which resistance mechanisms are already present, or likely to develop. The value of profiling both dimensions together is illustrated by p95HER2. A single receptor variant produces two concurrent failures: it lacks the epitope targeted by trastuzumab, impairing antibody binding and payload delivery, and it drives a PD-L1-inducing program that excludes cytotoxic T cells. The molecular and immunological consequences are therefore inseparable. While preclinical models suggest that the irreversible TKI neratinib can direct proteasomal degradation of the truncated receptor and restore T-DXd activity, how p95HER2 arises in human tumors is not fully established, and no data are available in patients treated with ADCs; consequently, it remains a priority for investigation in future research perspectives (70).

Such profiling leverages established analytical platforms through a more integrated approach. The pan-cancer “targetgram” framework proposed by Bosi et al. exemplifies this approach by displaying antigen expression alongside downstream determinants of payload activity in a single readout (126). Clinical relevance is concrete in the setting of sequential ADC therapy, where the efficacy of a subsequent ADC may be significantly influenced by payload class switching.

A real-world study in metastatic breast cancer addressed which ADC is most appropriate as a rechallenge after progression on a first ADC. It did not reach a definitive conclusion, but among the 40 patients who progressed on T-DM1, those switched to T-DXd rather than to disitamab vedotin had longer PFS2 (T-DXd 5.37 vs RC48 3.30 months, HR 0.40) and OS (T-DXd 50.6 vs RC48 20.2 months, HR 0.27, 95% CI 0.08–0.91). The disproportion between a modest PFS gain and a markedly larger OS difference may point to unmeasured confounding, yet the direction of effect is biologically coherent and warrants further investigation (127).

These conclusions are tempered by the nature of the available evidence. Most data derive from preclinical models, such as cell lines and xenografts that identify candidate mechanisms but cannot reproduce the complexity of human tumors. Moreover, several of the mechanisms catalogued here still require clinical validation. Key questions remain open: whether the target antigen only localizes the payload or also shapes the tumor’s interaction with the immune microenvironment; and which of several coexisting mechanisms should be addressed first. Their resolution will require translational endpoints embedded prospectively within trials.

Managing this complexity exceeds the reach of any single discipline. It requires multidisciplinary approaches that interact with one another, integrating clinical pharmacology, molecular pathology, clinical oncology, and other specialties to interpret a resistance profile that no single perspective can read alone. This integration is already emerging in real-world practice, but it must be consolidated.

## 8 Methods and limitations

This is a narrative review, organised around *a priori* conceptual framework rather than around the volume of available literature. The framework, the direct and immune-mediated pharmacology of ADCs, then the molecular, pharmacologic and immune-mediated determinants of resistance, then the therapeutic implications that follow from them, was defined before the literature was assembled; evidence was subsequently sought to populate, test and where necessary revise it. This design privileges mechanistic coherence over exhaustive coverage, and the resulting risk of selection bias is addressed in this section.

Literature was identified in PubMed and Google Scholar, with no lower date limit, and extended by backward citation chasing from the reference lists of retrieved articles. Search concepts combined the drug class and each approved agent, the disease setting (breast cancer, HER2-positive, HER2-low and TNBC), and the biological process addressed in each section. Only English-language publications were considered.

Sources were retained on the basis of topical relevance and of a study design permitting attribution of the reported finding to a defined experimental or clinical observation. Where several reports addressed the same mechanism, the primary publication was cited in preference to subsequent reviews. Preclinical studies and data available only as conference reports are identified as such at the point of citation; ongoing trials are cited by registration number and reported for design and rationale only. Regulatory information was obtained from FDA and EMA labels and approval records. The literature search was conducted between October 2025 and June 2026.

The framework of this article was defined before the literature was assembled; mechanisms not anticipated by that framework may be underrepresented. Because resistance mechanisms found not to operate are seldom reported, the available literature is enriched for positive findings. Where mechanisms described in separate studies converge on a common consequence, we have grouped them accordingly, and such connections are interpretive rather than demonstrated. Most of the mechanisms discussed derive from small paired-biopsy series, from cell lines rendered resistant *in vitro*, or from animal models that cannot reproduce the immune context in which these drugs act, and several are supported by evidence from other tumour types or from unconjugated antibodies. Assays bearing the same name do not always measure the same analyte, and most mechanisms lack a validated clinical assay or threshold. None has undergone prospective validation, and for several candidate markers the reported predictive value differs between cohorts.
