# Supplementary material for: Molecular and immune-mediated resistance to antibody-drug conjugates in breast cancer
Source: Front Immunol. 2026 Oct 9;17:1927515. doi: 10.3389/fimmu.2026.1927515 (PMC13691731; doi:10.3389/fimmu.2026.1927515)
Supplement: Supplementary file 1 [file Table1.docx]

Supplementary Table 1. Mechanisms of resistance to antibody-drug conjugates in breast cancer: molecular basis, clinical evidence, and detection methods.

| **Mechanism** | **ADC affected** | | **Molecular description** | | **Evidence** | | **Detection method** | | |
| --- | --- | --- | --- | --- | --- | --- | --- | --- | --- |
| **Receptor-related resistance** | | | | | | | |  |  |
| HER2 downregulation / heterogeneity | T-DXd | Decline of HER2 protein expression between baseline and progression biopsy reduces ADC binding and payload delivery. | | **Clinical**: DAISY trial: paired baseline/progression biopsies analyzed in n=20 patients with HER2+ mBC on T-DXd; 13/20 (65%, 95% CI 40.8–84.6) showed decline in HER2 expression at progression (68). | | IHC + reflex ISH per ASCO/CAP–ESMO 2023 algorithm (validated clones 4B5, HercepTest; dual-probe ISH preferred) (69). | | | |
| p95HER2 (HER2 shedding via ADAM10/DPAGT1) | T-DXd,  trastuzumab *(T-DM1: not directly tested for resistance)* | ADAM10 cleaves HER2 generating a membrane-bound truncated form (p95HER2; lacks trastuzumab epitope, retains kinase activity) and a soluble HER2-ECD. | | **For ADCs (T-DXd):**  **Preclinical** **(in vitro + xenograft):** in preclinical models, p95HER2 impairs T-DXd efficacy through induction of an immunosuppressive program; neratinib has been shown to reverse this by directing proteasomal degradation of p95HER2 (70).  **For trastuzumab:**  **Clinical:** VeraTag p95 cutoff independently validated in 240 trastuzumab-treated MBC pts (HR 1.94 OS, p=0.0055) (71). | | **For ADCs (T-DXd):**  **preclinical only**: in vitro + xenograft, T-DXd efficacy impaired in p95HER2-expressing models; reversed by neratinib (70).  **For trastuzumab (non-ADC):**  VeraTag quantitative FFPE immunoassay (Monogram Biosciences) with anti-p95 clone D9; predefined cutoff prognostic on trastuzumab. No clinical assay yet validated to predict T-DXd response (71). | | | |
| TROP2 expression variability | SG | Spatial heterogeneity may reduce ADC binding to a fraction of tumor cells, allowing escape of TROP2-low subclone. | | **Clinical (ASCENT biomarker analysis, mTNBC; n=290 Trop-2-evaluable of 468 randomized; n=151 SG vs n=139 TPC):** lower TROP2 H-score associated with reduced SG efficacy. By 3-category H-score cut-off (0-100 low / 100-200 medium / >200 high): mPFS in SG arm 6.9 / 5.6 / 2.7 mo; ORR in SG arm 44% / 38% / 22% (72). Quartile-based reanalysis confirmed the trend: Q1 (H-score 0-130) mPFS 2.7 mo (SG) vs 1.5 mo (TPC) HR 0.583; Q3 (H-score 220-275) mPFS 6.8 mo (SG) vs 1.6 mo (TPC) HR 0.196 (73). SG retained PFS and OS benefit over TPC across all expression subgroups, including TROP2-low. | | IHC on FFPE with anti-TROP2 mouse monoclonal antibody (clone ENZ ABS380-0100, Enzo Life Science), OptiVIEW DAB detection kit; H-score thresholds 0<100 low / 100–200 medium / >200 high (72). Predictive but not actionable: SG benefit retained across high/medium expression levels in mTNBC. | | | |
| MUC4 upregulation (steric masking) | T-DM1,  trastuzumab | TNFα activates NF-κB signaling, upregulating MUC4. MUC4 is a transmembrane glycoprotein that sterically masks the trastuzumab epitope on HER2, reducing antibody binding. | | **For ADCs (T-DM1):**  ***in vitro*** **only**, silencing MUC4 (siRNA) overcame T-DM1 antiproliferative effects in BT-474 cells overexpressing TNFα (74).  **For trastuzumab:**  ***Clinical*:** 78 HER2+ pts adjuvant trastuzumab, MUC4 independent predictor of poor DFS, HR 5.43 (95% CI 1.56-18.80), p=0.008 (74).  ***Clinical:***  in 91 HER2+ trastuzumab-treated patients, MUC4 expression correlated with reduced tumor-infiltrating lymphocytes and associated with an immune-desert phenotype (75). | | IHC, clone 1G8 Santa Cruz (74). No standardized cut-off in breast cancer.  **Note:** alternative anti-MUC4 clone 8G7 (Moniaux et al. 2004). Proposed as a tool for MUC4 detection in solid tumors (76). | | | |
| TROP2 T256R missense mutation | SG | Aquired missense mutation in *TACSTD2*/TROP2 (T256R) causes defective plasma membrane localization, reducing cell-surface binding of the SG antibody. | | **Clinical,** **single patient case:** TROP2 T256R identified in multiple thoracic metastatic lesions of one mTNBC patient with acquired resistance to SG (77).  **in vitro:** BT549 cells reconstituted with wild-type vs T256R cDNA showed equivalent protein expression but defective membrane localization and reduced hRS7 binding (77). | | NGS (panel sequencing including TACSTD2; not in standard breast panels). | | | |
| Endophilin A2 (SH3GL1) loss | T-DM1 | Loss of endophilin A2, an endocytic adaptor required for HER2 internalization, impairs delivery of T-DM1 to lysosomes, and reduces cytotoxicity. | | **Clinical (associative, IHC + transcriptomic)*:*** Endo II protein highly expressed in HER2+ primary tumors and lymph node metastases by IHC; low SH3GL1 mRNA associated with longer relapse-free (n=146) and overall survival (n=56) in node-positive HER2+ breast cancer (78).  **In vitro (HER2+ breast):** stable silencing of Endo II in SK-BR-3 and HCC1954 increased surface HER2, reduced HER2 internalization, and suppressed T-DM1 cytotoxicity (79). | | IHC (research-grade, H-score) or *SH3GL1* mRNA expression; no validated clinical assay or cut-off for T-DM1 resistance prediction. | | | |
| Lipid raft / Caveolin-1 mediated trafficking alteration | T-DM1 | T-DM1 internalization is mediated by caveolin-1 in cholesterol-rich lipid rafts; perturbation of caveolar trafficking alters T-DM1 endocytosis and lysosomal delivery. | | **In vitro breast cell,** higher CAV-1 expression correlated with greater T-DM1 internalization and cytotoxicity (80); metformin-induced CAV-1 upregulation enhanced T-DM1 efficacy (80).  **In gastric cancer (N87 cells, Sung et al. 2018):** opposite finding — caveolin-1 and cavin-1 upregulated in T-DM1-resistant cells; T-DM1 colocalized with CAV-1 in intracellular puncta with reduced lysosomal trafficking. CAV-1 shRNA knockdown, however, did not re-sensitize resistant cells, leaving causality unproven (81). | | Research only, no clinical assay available | | | |
| SLC46A3 loss | T-DM1; *cross-resistance to other ADCs with non-cleavable maytansinoid or PBD payloads* | Loss of SLC46A3, a lysosomal membrane transporter, prevents the active catabolite lysine-MCC-DM1 from exiting the lysosome into the cytoplasm, blocking microtubule binding and cytotoxicity. | | **In vitro (shRNA screen in HER2+ breast cancer cell lines):** loss of SLC46A3 conferred resistance to multiple non-cleavable maytansinoid ADCs including T-DM1 (82). Sensitivity restored by SLC46A3 re-expression in refractory lines (83).  **In vitro + in vivo (xenografts, multiple tumor types):** low SLC46A3 expression identified as mechanism of both innate and acquired resistance to ADCs bearing DM1 and PBD (SG3376) payloads (83). | | mRNA expression measured by qRT-PCR or RNA-seq (the method used in the original studies). | | | |
| Lysosomal alkalinization + reduced proteolytic activity (cathepsin B) | T-DM1 *(T-DXd has GGFG linker cleaved by cathepsin B; potentially vulnerable to the same mechanism but not directly tested)* | Increased lysosomal pH reduces the activity of acidic proteases including cathepsin B, impairing proteolytic degradation of the trastuzumab moiety and trapping T-DM1 in the lysosome without release of the active catabolite lysine-MCC-DM1. | | **In vitro + in vivo:** in the resistant clones, T-DM1 entered the lysosome normally but remained trapped there without payload release; lysosomal pH was increased and cathepsin B activity was **significantly lower** identifying impaired lysosomal proteolysis as one of the mechanisms of the resistance (84). | | Research only; no clinical assay is available. | | | |
| **Payload efflux mechanisms** | | | | | | | | |  |
| MDR1 / ABCB1 (P-glycoprotein) upregulation | T-DM1 *(generalizable: ADCs with payloads substrate of MDR1)* | Increased expression of ABC family efflux transporters pumps the cytotoxic payload out of tumor cells, lowering intracellular drug concentration below the cytotoxic threshold. | | **Clinical (T-DXd, real-world retrospective cohort, n=2,799 mBC patients):**  in pre-treatment samples, higher ERBB2 and lower ABCC expression were associated with longer T-DXd OS (ABCC1 Q1-low 22.0 vs Q4-high 14.2 mo, p<0.0001); ABCC1 predicted OS independently of HER2 and was predictive not prognostic (no effect in a treatment-agnostic cohort, HR 1.03, p=0.152).  Post-T-DXd samples (compared to unmatched T-DXd-naïve samples) were enriched for mutations in *ERBB2*, *NFE2L2* (transcriptional activator of *ABCC1*), and *KEAP1* (negative regulator of *NFE2L2*) mutations, predicted to upregulate ABCC1 (85).  **In vitro (cell lines):** KPL-4 T-DM1-resistant clone (KPL-4 TR) showed strong MDR1 upregulation and contributing to T-DM1 resistance; MDR1 inhibitor (XR9051) restored sensitivity (86). | | mRNA expression by qRT-PCR, RNA-seq, or commercial transcriptomic profiling; IHC with research-grade antibodies also reported. No cut-off for ADC resistance prediction in breast cancer. | | | |
| Defective Cyclin B1 induction | T-DM1 | In sensitive cells, T-DM1 induces cyclin B1 accumulation, which activates CDK1 and drives a G2–M arrest culminating in mitotic catastrophe. Resistant cells do not induce cyclin B1, evade the G2–M arrest, and escape apoptosis. | | **Ex vivo (human tissue):** in 18 HER2+ breast cancer fresh explants cultured with T-DM1, cyclin B1 was induced in 12/18 (66.6%) (87).  **In vitro:** three T-DM1-resistant cell lines showed defective cyclin B1 siRNA knockdown conferred resistance in all three parental lines, and cdc20 silencing (raising cyclin B1) partially restored sensitivity in 2/3 resistant lines (87). | | IHC on FFPE (antibody sc-245, Santa Cruz Biotechnology). Research only, no clinical assay available | | | |
| TOP1 mutations (acquired) | *all ADCs with TOP1 inhibitor payloads (SN-38 or DXd derivatives)* | Acquired somatic missense mutations in *TOP1* alter the enzyme's catalytic activity and DNA binding, reducing the formation of stable TOP1-DNA cleavage complexes that camptothecin-derived payloads (SN-38, DXd) require to exert cytotoxicity. | | **Clinical (cohort, mBC):** 4/31 (12.9%) ADC-treated pts developed TOP1 mutations (S57C, R364H, W401C, G359E) at progression vs 0.7% (3/420) non-ADC (88).  **Case report** **(mTNBC, autopsy):** TOP1 E418K + frameshift mutations identified at progression in 1 SG-resistant patient. Distinct metastatic lesions harbored TROP2 T256R, indicating parallel resistance affecting both payload and antibody targets (77). | | Liquid biopsy ctDNA NGS panel including *TOP1* (e.g., GuardantOMNI 500-gene), or tumor tissue NGS. *TOP1* is not included in most standard breast cancer NGS panel. | | | |
| PLK1 overexpression | T-DM1; *potentially other ADCs with anti-microtubule payloads (MMAE, MMAF); not directly tested* | High PLK1 expression allows resistant cells to bypass the mitotic checkpoint that T-DM1 activates by disrupting microtubules. Pharmacological inhibition of PLK1 (volasertib) restores the checkpoint and re-sensitizes cells to T-DM1. | | **Clinical correlation (gene expression, indirect):** T-DM1 resistance signature and PLK1 expression predict lower sensitivity to taxane + trastuzumab in HER2+ breast cancer patients (89).  **In vivo (cell line xenograft):** combination of volasertib + T-DM1 produced synergistic tumor growth inhibition compared to either agent alone (89).  **In vitro (HER2+, breast):** siRNA sensitization screen in acquired (BT-474/SK-BR-3 T-DM1R) and de novo (JIMT-1) models identified PLK1 as a resistance mediator; the authors conclude a high dependency of T-DM1 resistance on PLK1 overexpression, whose inhibition restores the T-DM1-associated phenotypes (mitotic arrest, apoptosis, DNA damage) (89). | | **PLK1 mRNA by RNA-seq or qRT-PCR; protein by Western blot.** Research-grade only, no validated assay or cut-off for ADC resistance prediction in breast cancer. | | | |
| CCNE1 amplification | T-DXd | Cyclin E1 overexpression hyperactivates CDK2 and forces premature S-phase entry with replication stress; in this state, T-DXd-induced DNA damage is tolerated rather than fatal, and cells survive payload exposure. | | **In vitro (HER2+ breast and other):** doxycycline-induced cyclin E overexpression decreased T-DXd sensitivity; cyclin E knockdown increased sensitivity (90). | | Cyclin E1 IHC (research-grade). Research only; no clinical assay is available. | | | |
| SLX4 mutations (acquired) | T-DXd | SLX4 is a DNA repair scaffolding protein involved in resolving DNA damage induced by topoisomerase I inhibitors. Mutations enriched in post-T-DXd biopsies are predicted to impair DNA damage repair, allowing cells to survive DXd-induced damage. | | **Clinical (DAISY trial, breast):** 14% (3/21 pts.) of post-progression biopsies harbored SLX4 mutations (incl. M1591L) (68).  **In vitro:** SLX4 siRNA knockdown in SK-BR-3 (HER2+ breast) and MCF-7 cell lines reduced DXd payload sensitivity (68). | | NGS panel sequencing including SLX4. | | | |
| **Apoptosis resistance** | | | | | | | | |  |
| Bcl-2 / Bcl-xL upregulation | T-DM1 *(generalizable: anti-apoptotic axis)* | When T-DM1 releases its payload, the cell normally triggers apoptosis through the intrinsic pathway; high baseline BCL-2/BCL-XL expression raises the apoptotic threshold, blocking this response and allowing cells to survive payload exposure. | | **In vivo (5 PDX, HER2-expressing breast):** ABT-263 (navitoclax) enhanced T-DM1 cytotoxicity in 3/5 PDX, including 2 HER2-low/ERBB2-non-amplified models. Effect mediated mainly by BCL-XL (91).  **Note**: the 2 most responsive PDX expressed HER2-low protein and lacked ERBB2 amplification. | | BCL-2 and BCL-XL IHC; intensity scored qualitatively as negative (0), weak (1+), moderate (2+), or strong (3+), reported as H-score. Research only; no clinical assay is available. | | | |
| TACC3 overexpression | T-DM1 | In sensitive cells, T-DM1-induced microtubule disruption triggers mitotic arrest, immunogenic cell death (ICD), and priming of anti-tumor CD8+ T cells. In resistant cells, TACC3 overexpression stabilizes the mitotic spindle, bypassing the spindle assembly checkpoint and abolishing ICD. | | **Clinical (HER2+, T-DM1-treated; 57 patients / 71 samples):** Cross-sectional group comparison: frequency of TACC3-high tumors rose in resistant (48%→64%, pre.→post.) and fell in sensitive (53%→17%). In the same cohort, post-treatment CD8-high frequency was higher in sensitive than resistant tumors (75% vs 43%) (40).  **In vivo (murine HER2+):** TACC3 inhibition + T-DM1 induced dendritic cell maturation, intratumoral CD8+ infiltration, and ICD in vaccination assay (40).  **In vitro (HER2+ breast):** TACC3 overexpressed in T-DM1-resistant SK-BR-3 and BT-474 cells; genetic/pharmacologic TACC3 inhibition restored SAC activation and ICD markers (40). | | TACC3 IHC on FFPE (antibody validation performed using normal breast as negative and IDC tumor as positive control). No validated clinical assay or cut-off for ADC resistance prediction in breast cancer. | | | |
| Cancer-associated fibroblasts (CAFs): stromal barrier and resistance-associated subsets | T-DXd, T-DM1, TROP2-ADCs | CAFs contribute to ADC resistance through two non-mutually-exclusive mechanisms:  (i) FAP+ activated CAFs form a fibrotic barrier reducing antibody and payload uptake (92);  (ii) a PDGFRa+/FAP- CAF subset is enriched at residual disease specifically in T-DM1-treated patients (93). | | **Clinical (PREDIX HER2 trial, neoadjuvant breast, 57 patients):** in HER2+ tumors treated with T-DM1, high baseline density of a specific CAF subset (PDGFRa+/FAP-) predicted residual disease at surgery. The effect was specific to T-DM1 and was not observed in the other arm suggesting these CAFs drive resistance to the ADC payload, not to anti-HER2 therapy in general.) (93).  **In vivo (9 PDX, cross-tumor incl. breast):** T-DXd low-response PDX showed higher FAP+ area and fibrosis vs high-response, with no difference in HER2 (92).  ***In vivo* (mouse CDX, MDA-MB-468 TNBC breast):** FAK inhibitor + TROP2-ADC outperformed either monotherapy (92). | | Multiplex immunofluorescence (in situ), stromal panel incl. PDGFRα + FAP (PREDIX HER2 trial). nNo validated clinical assay Or cut-off for ADC resistance prediction in breast cancer. | | | |
| MMP1 upregulation (NF-κB-driven EMT) | SG | In sensitive HR+ breast cancer cells, SG payload (SN-38) induces DNA damage and apoptosis. In SG-resistant cells, MMP1 overexpression activates the NF-κB pathway, drives an EMT phenotype (loss of E-cadherin, gain of N-cadherin and vimentin), and reduces sensitivity to the SN-38 payload. | | **Clinical: IHC on patient tumors, MMP1 expression was approximately 3.5x higher in SG-resistant than SG-sensitive cases (94).**  **In vivo (CDX, HR+ breast):** MMP1 knockdown + SG slowed tumor growth more than SG alone (94).  **In vitro (HR+ breast):** in MCF-7 and T47D resistant lines, MMP1 knockdown restored SG sensitivity (94). | | MMP1 IHC on FFPE (Abcam ab52631). No validated clinical assay or cut-off for ADC resistance prediction in breast cancer. | | | |
| CD47 upregulation (impaired ADCP) | T-DXd | DXd payload upregulates surface CD47 ("don't eat me" signal) on HER2+ breast cancer cells, dampening macrophage-mediated phagocytosis (ADCP) and downstream CD8+ T-cell priming. DM1 has the opposite effect on CD47. | | **In vitro (HER2+ breast):** In several cell lines, T-DXd treatment increased CD47 expression. CD47 knockout restored ADCP, macrophage activation, inflammatory cytokine release, and CD8+ T-cell antigen presentation upon T-DXd (34). | | CD47 by cytometry. No validated clinical assay or cut-off for ADC resistance prediction in breast cancer. | | | |
| FCGR3A 158 V/F polymorphism → impaired ADCC | Trastuzumab *(ADC-specific impact: not investigated)* | The FCGR3A V158F polymorphism modifies the binding affinity of CD16 (FcγRIIIa) on NK cells for the IgG1 Fc region. F/F homozygotes show lower affinity and reduced ADCC compared with V/V. | | **In vitro (ADC, ADCC retention):** T-DM1 and T-DXd retain trastuzumab-equivalent ADCC activity against HER2+ cells (21, 28).  **Background (trastuzumab non-ADC, clinical):** in HER2+ metastatic BC, V/V genotype was associated with better ORR and PFS than F/F (Musolino et al. 2008, n=54 (95); Abdel-Wahed et al. 2024, n=80 (96)).  In larger adjuvant trials, evidence is discordant: in NSABP B-31 (n=1251 pts.), F/F homozygotes derived less benefit from trastuzumab (Gavin et al. 2017)(97); in NCCTG N9831 (n=1325 pts.), no significant predictive effect of FCGR3A genotype was observed (98). | | TaqMan SNP genotyping (rs396991) or NGS panel. | | | |
